# *Toxoplasma gondii* infection: relationship between seroprevalence and risk factors among primary schoolchildren in the capital areas of Democratic Republic of São Tomé and Príncipe, West Africa

**DOI:** 10.1186/1756-3305-5-141

**Published:** 2012-07-13

**Authors:** Chia-Kwung Fan, Lin-Wen Lee, Chien-Wei Liao, Ying-Chieh Huang, Yueh-Lun Lee, Yu-Tai Chang, Ângela dos Santos Ramos José da Costa, Vilfrido Gil, Li-Hsing Chi, Takeshi Nara, Akiko Tsubouchi, Olaoluwa Pheabian Akinwale

**Affiliations:** 1 Department of Parasitology, College of Medicine, Taipei Medical University, 250 Wuxing St, Taipei, 11031, Taiwan; 2 Center for International Tropical Medicine, College of Medicine, Taipei Medical University, 250 Wuxing St, Taipei, 11031, Taiwan; 3 Department of Microbiology and Immunology, College of Medicine, Taipei Medical University, 250 Wuxing St, Taipei, 11031, Taiwan; 4 Taiwan Medical Mission in São Tomé, São Tomé, C.P. 839, Democratic Republic of São Tomé and Príncipe; 5 Ministry of Health and Social Affairs, São Tomé, C.P. 23, Democratic Republic of São Tomé and Príncipe; 6 National Global Fund Program in Sao Tome, Sao Tome, C.P. 23, Democratic Republic of São Tomé and Príncipe; 7 Department of Molecular and Cellular Parasitology, Juntendo University School of Medicine, Tokyo, 113-8421, Japan; 8 Nigerian Institute of Medical Research, Lagos, P.M.B 2013, Nigeria

**Keywords:** Seroepidemiology, *Toxoplasma gondii*, Primary schoolchildren, Democratic Republic of São Tomé and Príncipe, West Africa

## Abstract

**Background:**

The status of *Toxoplasma gondii* infection among primary schoolchildren (PSC) of the Democratic Republic of São Tomé and Príncipe (DRSTP), West Africa, remains unknown to date.

**Methods:**

A serologic survey and risk factors associated *T. gondii* infection among PSC in the DRSTP was assessed by the latex agglutination (LA) test and a questionnaire interview including parents’ occupation, various uncomfortable symptoms, histories of eating raw or undercooked food, drinking unboiled water, and raising pets, was conducted in October 2010. Schoolchildren from 4 primary schools located in the capital areas were selected, in total 255 serum samples were obtained by venipuncture, of which 123 serum samples were obtained from boys (9.8 ± 1.4 yrs) and 132 serum samples were obtained from girls (9.7 ± 1.3 yrs).

**Results:**

The overall seroprevalence of *T. gondii* infection was 63.1% (161/255). No significant gender difference in seroprevalence was found between boys (62.6%, 77/123) and girls (63.6%, 84/132) (*p* = 0.9). The older age group of 10 years had insignificantly higher seroprevalence (69.9%, 58/83) than that of the younger age group of 8 year olds (67.7%, 21/31) (*p* = 0.8). It was noteworthy that the majority of seropositive PSC (75.8%, 122/161) had high LA titers of ≥1: 1024, indirectly indicating acute or repeated *Toxoplasma* infection. Parents whose jobs were non-skilled workers (73.1%) showed significantly higher seroprevalence than that of semiskilled- (53.9%) or skilled workers (48.8%) (*p* < 0.05). Children who had a history of raising cats also showed significantly higher seroprevalence than those who did not (*p* < 0.001).

Children who claimed to have had recent ocular manifestation or headache, i.e. within 1 month, seemed to have insignificantly higher seroprevalence than those who did not (*p* > 0.05).

**Conclusions:**

Parents’ educational level and cats kept indoors seemed to be the high risk factors for PSC in acquisition of *T. gondii* infection. While, ocular manifestation and/or headache of PSC should be checked for the possibility of being *T. gondii* elicited. Measures such as improving environmental hygiene and intensive educational intervention to both PSC and their parents should be performed immediately so as to reduce *T. gondii* infection of DRSTP inhabitants including PSC and adults.

## Background

Although toxoplasmosis is a cosmopolitan infection, the disease appears to be overshadowed in the tropics by other endemic diseases such as malaria and HIV [1]. *Toxoplasma gondii* is a widespread protozoan parasite whose definitive host is the cat. Human infection mainly occurs via close contact with the soil or accidental ingestion of contaminated water or food by *Toxoplasma* oocysts excreted in cat feces [2]. Upon acquiring *T. gondii* infection, immunocompetent adults and children are usually asymptomatic or have spontanesouly resolved symptoms such as fever, malaise, and lymphadenopathy indicating thus, a symptomless latent infection [3]. Among immuno-compromised individuals, e.g. people with acquired immunodeficiency syndrome (AIDS), *T. gondii* causes severe encephalitis via the acute infection or reactivation of latent infection [4,5]. Moreover, for pregnant women, newly acquired *T. gondii* infection can be transmitted to the fetus causing mental retardation, blindness, epilepsy, and death. Although congenital toxoplasmosis may be asymptomatic at birth, ocular problems can manifest later in life [6].

Infection by the protozoan parasite *T. gondii* is widely prevalent in animals and humans worldwide [7]. Regarding *T. gondii* infection diagnosis, organism detection is rarely achievable. Thus, most clinical laboratories use serological tests to detect antibodies against *T. gondii*, such as the latex agglutination (LA) test, ELISA, and IFAT. Due to its high specificity and sensitivity as well as convenience, the LA test has been widely used in remote areas in developing countries [4,8-13]. In developed countries, the prevalence of *Toxoplasma* infection among primary schoolchildren (PSC) was low with a range of 0.0% to 11.0% in Japan and Ireland, respectively [14,15]. In Asia, East Europe, and Latin American, the seroprevalence in Iran, Indonesia, Slovak, and Brazil was high, ranged from 20.9% to 68.4% [16-18]. In Africa, studies concerning the seroprevalence of *T. gondii* infection in PSC have been limited; the seroprevalence of *T. gondii* infection in PSC was 37.5%, 36.3%, and 0.0% in Somalia, East Africa, Madagascar off the Southeastern coast of Africa, and Swaziland in Southern Africa, respectively [13,19,20]. Although our previous study found that the seroprevalence of *T. gondii* infection among DRSTP pre-schoolchildren under 5 yrs old was not low, reaching 21.5% (26/121) [12], information related to *T. gondii* infection among DRSTP PSC remains largely unknown to date.

In this study, we examined *T. gondii* antibody titers of 255 PSC from 4 primary schools located in the capital areas of DRSTP using the LA test conducted in October 2010. The study may help the DRSTP delineate effective measures against toxoplasmosis to improve the health status of DRSTP children.

## Methods

### Geographic description, study population, and subject selection

**Figure 1:**
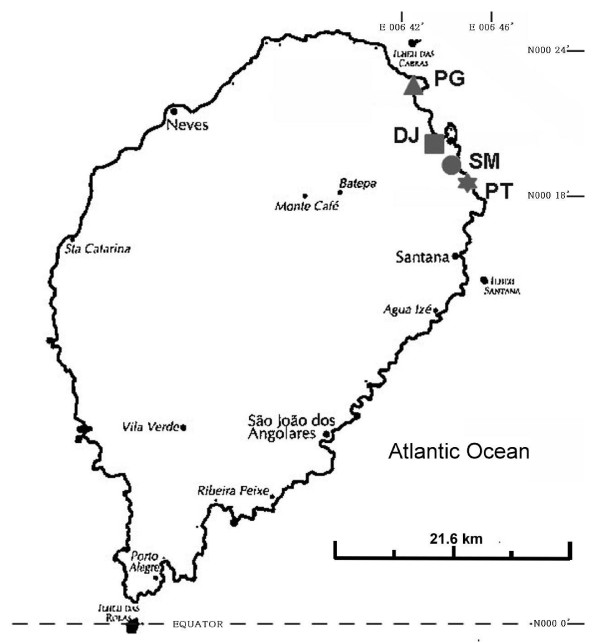
Map indicating the geographic location of 4 primary schools (SM, PT, PG, and DJ) located in capital areas of the Democratic Republic of São Tomé and Príncipe, West Africa.

The DRSTP consists of the two islands of São Tomé and Príncipe and a number of smaller islets in the Gulf of Guinea. São Tomé lies approximately 180 miles from Gabon on the West African coast, and the equator crosses its southern tip. The climate is tropical with two rainy seasons. The total number of inhabitants in the DRSTP is estimated to be 160,000, and the total number of inhabitants on São Tomé Island is approximately 150,000. This study was conducted on 6 ~ 24 October 2010. Forty to eighty schoolchildren of grades 4 and 5 (with a mean age ± SD of 9.8 ± 1.3 yr) from 4 public primary schools each (SM, PT, PG, and DJ) which were located in the capital areas (Figure 1) were selected for enrollment in the present study according to suggestions of the Ministry of Health and Social Affairs after informed consent was obtained from parents, guardians, or school representatives. All of the 4 schools have similar geographical characteristics and were close to the seashore.

Each subject completed a questionnaire and was interviewed by trained public nurses and/or an assistant. Sociodemographic information was obtained directly from each individual through a questionnaire that requested various personal details, including age, sex, weight, height, parents’ occupation, and residential district. In addition, items regarding whether the PSC had uncomfortable symptoms such as ocular manifestation, headache, abdominal pain, or seizure as well as histories of eating raw or undercooked meat (including pork, beef, goat and chicken) or vegetables, drinking unboiled water, and raising pets (cats and dogs) were also included in the questionnaire.

### Latex agglutination test (LA)

In this study, all sera were screened usin g the *Toxoplasma* latex agglutination test employing commercial reagents (TOXO Test-MT, Eiken Co. Ltd, Tokyo, Japan). Compared to the Sabin-Feldman test, the sensitivity and specificity of this test were 96.3% and 97.1%, respectively [8]. The test was performed according to the manufacturer’s instructions, with the help of a 96-well U-form microtiter plate (Nerbe, Germany), buffer solution, and latex solution. Sera found positive at titers 1/32 (*i. e.*, 1:32 to 1:2048) were regarded as positive [12].

In total, 255 serum samples were obtained by venipuncture, of which 123 serum samples from boys and 132 serum samples from girls were randomly collected from apparently healthy PSC. The mean ages were similar in both genders (boys at 9.8 ± 1.4 yr vs*.* girls at 9.7 ± 1.3 yrs). All serum specimens were kept at −20°C at the Parasitology Laboratory in Central Hospital of the DRSTP until the laboratory examination.

### Ethical approval

Ethical approval for the study was obtained from the Ministry of Health and Social Affairs of the Democratic Republic of São Tomé and Príncipe.

### Statistical analysis

In the present study, the subjects were categorized into 4 age groups (8-yr-old, 9- yr-old, 10-yr-old, and 11-yr-old group). Serum samples that showed LA positivity were considered seropositive. Statistical analyses were performed using SPSS software (SPSS, Chicago, IL, USA). Crude odds ratios (ORs) with their 95% confidence intervals (CI) were estimated and *p* values of < 0.05 were considered statistically significant.

## Results

Of the total 255 serum samples studied, 161 (63.1%; 161/255) were positive for *Toxoplasma* antibody as determined by LA test (Table 1). No significant gender difference in seroprevalence was found between boys (62.6%, 77/123) and girls (63.6%, 84/132) (OR = 1.1, 95% CI =0.6–1.7, *p* = 0.9). The older age group of 10 years had insignificantly higher seroprevalence (69.9%, 58/83) than that of the younger age group of 8 year olds (67.7%, 21/31) (ORs = 1.1, 95% CI = 0.5–2.7, *p* = 0.8) (Tables 1 and 2). Also, no significant difference in seroprevalence between schools of SM (58.9%, 43/73), PT (65.3%, 49/75), PG (67.5%, 27/40), and DJ (58.9%, 42/67) was found (*p* > 0.05). The overall LA titer distributions in seropositive PSC were 1:32 (0.0%, 0/161), 1:64 (0.0%, 0/161), 1:128 (1.2%, 2/161), 1:256 (7.2%, 12/161), 1:512 (15.5%, 25/161), 1:1024 (19.3%, 31/161), 1:2048 (14.3%, 23/161), and 1: 4096 (42.2%, 68/161), respectively. In boys, the LA titer distributions were 1:32 (0.0%, 0/77), 1:64 (0.0%, 0/77), 1:128 (1.3%, 1/77), 1:256 (7.8%, 6/77), 1:512 (16.9%, 13/77), 1:1024 (16.9%, 13/77), 1:2048 (12.9%, 10/77), and 1: 4096 (44.2%, 34/77), respectively. In girls, the LA titer distributions were 1:32 (0.0%, 0/84), 1:64 (0.0%, 0/84), 1:128 (1.2%, 1/84), 1:256 (7.1%, 6/84), 1:512 (14.3%, 12/84), 1:1024 (21.4%, 18/84), 1:2048 (21.4%, 13/84), and 1: 4096 (40.5%, 34/84), respectively. It was noteworthy that the majority of seropositive PSC (75.8%, 122/161) had high LA titers of ≥1: 1024, indirectly indicating acute or repeated *Toxoplasma* infection.

**Table 1:** **Demographic characteristics of the seroprevalence of*Toxoplasma gondii*antibody among primary schoolchildren in the Democratic Republic of São Tomé and Príncipe**

**Variable**	**Group**	**No. tested**	**No. positive**	**Percentage (%)**
**Gender**	Boys	123	77	62.6
	Girls	132	84	63.6
**Age group (yr)**	8 ≦	31	21	67.7
	9	82	52	63.4
	10	83	58	69.9
	^≧ 11^	59	30	50.9
**Parents Occupation**	Skilled worker	41	20	48.8
	Semiskilled worker	65	35	53.9
	Non-skilled worker	145	106	73.1
**Symptoms**	Ocular			
	No	123	77	62.6
	Yes	39	28	71.8
	Headache			
	No	61	35	57.4
	Yes	176	114	64.8
	Abdominal pain			
	No	50	36	72.0
	Yes	190	117	61.6
	Seizure			
	No	134	86	64.2
	Yes	6	3	50.0
**Risk factors**	Raising dogs			
	No	100	61	61.0
	Yes	139	94	67.6
	Raising cats			
	No	115	0	0.0
	Yes	126	123	97.6
	Playing in the soil			
	No	4	2	50.0
	Yes	66	43	65.2
	Eating raw meat			
	No	4	4	100.0
	Yes	66	65	98.5
	Eating raw vegetables			
	No	23	16	69.6
	Yes	223	142	63.7
	Drink unboiled water			
	No	52	31	59.6
	Yes	194	127	65.5
Total		255	161	63.1

**Table 2:** **Crude odds ratios (ORs) with 95% confidence intervals (CI) for various risk factors associated with seropositivity of*Toxoplasma gondii*antibodies among primary schoolchildren in the Democratic Republic of São Tomé and Príncipe**

**Variable**	**Group**	**ORs (95% CI)**	***p* value**
**Gender**	Boys	Referent	
	Girls	1.1 (0.6–1.7)	0.9
**Age group (yr)**	8	Referent	
	9	0.8 (0.3–2.0)	0.9
	10	1.1 (0.5–2.7)	0.8
	^≧ 11^	0.5 (0.2–1.2)	0.1
**Parents jobs**	Skilled worker	Referent	-
	Semiskilled worker	1.2 (0.6–2.7)	0.6
	Non-skilled worker	2.9 (1.4–5.8)	0.03
**Symptoms**	Ocular		
	No	Referent	-
	Yes	1.5 (0.7–3.3)	0.3
	Headache		
	No	Referent	-
	Yes	1.4 (0.8–2.5)	0.3
	Abdominal pain		
	No	Referent	-
	Yes	0.6 (0.3–1.2)	0.2
	Seizure		
	No	Referent	-
	Yes	0.6 (0.1–2.9)	0.5
**Risk factors**	Raising dogs		
	No	Referent	-
	Yes	1.3 (0.8–2.3)	0.3
	Raising cats		
	No	Referent	-
	Yes	-	< 0.001
	Playing in the soil		
	No	Referent	-
	Yes	1.9 (0.3–14.2)	0.5
	Eating raw meat		
	No	Referent	-
	Yes	-	0.8
	Eating raw vegetables		
	No	Referent	-
	Yes	0.8 (0.3–1.9)	0.8
	Drink unboiled water		
	No	Referent	-
	Yes	1.3 (0.7–2.4)	0.4

Parents whose jobs were non-skilled workers (73.1%) showed significantly higher seroprevalence than that of semiskilled (53.9%) and skilled workers (48.8%) (ORs = 1.2, 2.9; 95% CI = 0.6–2.7, 1.4–5.8; *p* = 0.6, 0.03, respectively). Children who had a history of raising cats (97.6%, 123/126) also showed significantly higher seroprevalence than those who did not (0.0%, 0/100) (*p* < 0.001). Children who had recently reported ocular manifestation (71.8%, 28/39) or headache (64.8%, 114/176) within 1 month seemed to have insignificantly higher seroprevalence than those who did not (62.6%, 77/123; 57.4%, 35/61) (ORs = 1.5, 1.4; 95% CI = 0.7–3.3, 0.8–2.5; *p* = 0.3, 0.3, respectively) (Tables 1 and 2).

## Discussion

Primary schoolchildren are particularly vulnerable to toxoplasmosis due to their habits of playing in water, soil, eating various raw foods, or contact with pets, including dogs and cats and hence they are an ideal target group to investigate toxoplasmosis prevalence. Data collected from this age group can thus be used to assess whether toxoplasmosis threatens the health of school-aged children, and also as a reference for evaluating the need for community interventions [21].

DRSTP is a tropical developing country; climatic and living conditions favor the surveillance of many parasites [22], including *T. gondii*. However, systemic studies concerning prevalence of *T. gondii* infection in the DRSTP PSC remains largely unclear to date. The present study is the first report indicating that the overall seroprevalence of *T. gondii* infection among PSC, living in capital areas of the DRSTP was indeed high, reaching 63.1%. However, this figure was higher than that reported in similar ages of schoolchildren in Iran (20.9%) and Indonesia (50.0%), Slovakia (20.5%),, Somalia (37.5%), Madagascar (36.3%) Nigeria (23.8%), and Swaziland (0.0%), but showed a similar prevalence to Brazil (68.4%) [13,16-20,23]. The discrepancy in seroprevalence in different countries may be due to different ethnicity, traditional culture, and food habits [7,24].

The present study indicated that the parents’ occupation was a factor affecting the likelihood of primary schoolchildren becoming infected by *T. gondii*. Particularly, children whose parents’ occupations were non-skilled e.g., a labourer or farmer, had significantly higher seroprevalence than those whose jobs were skilled e.g., teacher. One explanation may be that children whose parents’ occupations are those of a non-skilled worker, may not have enough knowledge about personal hygiene so as to instruct their children, thus increasing the possibility of *T. gondii* infection to their children. A similar situation was also found in Nigeria, where it was reported that a farmer or livestock worker had a higher probability of acquiring *T. gondii* infection than a businessman or civil servant [25].

It is generally known that no gender difference is usually found in *T. gondii* prevalence [3], which the present study also confirmed. High LA titers of anti-*Toxoplasma* antibodies might be regarded as predictive of the occurrence of either acute infection [26,27] or reinfections of toxoplasmosis [28]. In the present study, high *Toxopla sma* LA titers (≥ 1: 1024) were found in the most of seropositive PSC including both boys and girls. It might be due to boys or girls having similar routes for acquisition of *T. gondii* infection through frequent contact with risk factors e.g., raising dogs, cats, playing in the soil, eating raw/undercooked meats, eating raw vegetables, and drinking unboiled water, thus leading to increased opportunity of newly acquired *T. gondii* infection through constant exposure to *T. gondii* oocysts and/or tissue cysts [29].

It is acknowledged that the seroprevalence increases with age as shown in data from various countries [1,10,11,28]. A hypothesis would be that the increase is a reflection of increasing ‘exposure years’ as the children get older [15]. However, in this study, a higher seroprevalence was observed among ≥ 8 year-old children as compared to older children aged more than 11 years old, however, the difference was not statistically significant. The reason for the decline in seroprevalence with age is not clear. This may be due to the sample number of each age group, which could be too small to show the discrepancy in seroprevalence versus age. It has been established that *Toxoplasma* infection is prevalent worldwide in man and animals but the frequency of infection varies from one country to another. This variation is presumably due to the presence or absence of cats or dogs, climatic factors, playing in the soil, and consumption of raw or improperly cooked meat or vegetables, or unboiled water [3,29].

However, meat consumption for the DRSTP people is not easy and is infrequent due to economic problems. In this study, we found raising cats seemed to be a predominate risk factor for children to acquire *T. gondii* infection, as revealed by questionnaire analysis. Considering the suitable climatic conditions for sporulation of *Toxoplasma* oocysts in tropical regions e.g., DRSTP [7], it seemed likely that exposure to cat feces or contact with soil or water contaminated by *Toxoplasma* oocysts was one of the most important factors associated with *Toxoplasma* infection in the childhood life due to children living and playing very close to the soil and water. Such a route of transmission would explain the similar incidences of seropositivity between boys and girls in the DRSTP. The present findings also re-support our previous study indicating *Toxoplasma* oocysts may be the predominant infection source to DRSTP pregnant women [4]. A similar infection route of oocyst ingestion was found for children living in urban in Brazil [30].

Noteworthy, is that children who claimed to have headache or ocular discomfort within one month, showed higher seroprevalence than those who did not. Substantial evidence has indicated that a high association exists between *T. gondii* infection and headaches. It is postulated that the parasite may be responsible for the neurogenic inflammation thought to cause different types of headaches [31,32]. In addition, many studies indicate that *T. gondii* may circulate in the blood of immunocompetent individuals and that parasitaemia could be associated with the reactivation of the ocular disease as well as toxoplasmosis, which has been reported to contribute a significant burden to eye disease in the United States [33,34]. Since DRSTP children have been exposed to *T. gondii* infection in very early life, as evidenced by seroprevalence (21.5%, 26/121) of children aged under 5-years old [12], there is therefore, an urgent need to examine their cerebral and ocular conditions to find the possible associations as to whether *T. gondii* infection contributes to such uncomfortable syndromes. Recently, a study indicated that infection with *Toxoplasma* may cause abdominal hernia that should be also seriously considered in those kids [35].

Our study also has some limitations such as the small number of subjects studied and indistinguishable acute or chronic *Toxoplasma* infection due to lack of immunological assays for serum anti-IgG or IgM Abs. More participants and qualified technicians as well as advanced ELISA diagnostic equipment are required so as to improve the quality of similar studies in the DRSTP in the future.

## Conclusion

Taken together, the high prevalence of infection documented in this study thus indicates a need to enforce methods of control and preventive measures against *Toxoplasma* infection in the DRSTP PSC. Maintaining good personal hygiene and consumption habits are definitely important concepts for school-aged children in avoidance of *T. gondii* infection. Educational intervention is also a very practical measure to instruct schoolchildren on the infection route so as to protect themselves from *T. gondii* infection. Parents as well as teachers play a very important role in instructing children to keep good personal hygiene and consumption habits that are all important measures to further minimize *T. gondii* infection to DRSTP PSC.

## Competing interests

The authors declare that they have no competing interests.

## Authors’ contributions

CKF, TN, CWL, YTC, OPAk, and LHC participated in the conception and design of the study; LWL, ASRJdaC, VG, YLL, and AT participated in the analysis and interpretation of data; CKF drafted and revised the article; and CKF gave final approval of the version to be published. All authors read and approved the final version of the manuscript.
